# Celebration of the 150th birthday of Professor Sunao Tawara

**DOI:** 10.1002/joa3.12914

**Published:** 2023-09-15

## CELEBRATION OF THE 150TH BIRTHDAY OF PROFESSOR SUNAO TAWARA

Kan Takayanagi, MD, PhD, Honorary Professor of Dokkyo Medical University, Honorary Director, Kasukabe Kosei Hospital (k‐takayanagi@skmg.jp)

Kazuo Matsumoto, MD, PhD, Editor in Chief, Journal of Arrhythmia

Professor Sunao Tawara was born in Oita, Japan on 5 July 1873 and first documented the presence of the atrioventricular node in 1906. Before his publication, Purkinje had found a special tissue in the ventricle and His had reported the presence of a fiber just proximal to the Purkinje fiber. Tawara was able to accurately describe the connection between the atrium and the ventricle using the sheep heart and summarized the whole system as the conduction system of the heart.

Last year, we held a commemorative symposium in Yokohama on the occasion of the 150th anniversary of Professor Sunao Tawara at the 68th JHRS Annual Meeting. In this special issue, we have received two additional messages from the University of Marburg, one from the Dean of the Faculty of Medicine and another from the Department of Pathology, where Tawara studied from 1903 to 1906 under the supervision of Professor Aschoff.

His publication on the conduction system of the heart was translated into Japanese in 1990 and into English in 2000. We hope that this special issue will be of value to clinicians and researchers throughout the world. This was the strong will of the late Prof. Kozo Suma (1900–2019) as documented by Prof. M Shimada.

## MESSAGE FROM THE FEDERAL REPUBLIC OF GERMANY

Dr. Martin Pohl

Hanover (2022), martinpohl@gmx.net

Former Counsellor (2022), Labour and Health Affairs, Embassy of the Federal Republic of Germany, Tokyo

Dear Professor Takayanagi and members of the Japanese Heart Rhythm Society

It gives me great pleasure to hear that you are publishing a commemorative publication to mark the 150th birthday of Professor Sunao Tawara. As a former diplomat at the German Embassy in Tokyo, responsible for labor and health, and as a former Associate Professor of Management at the University of Tsukuba, I worked in Japan for 17 years and still have close ties with the country, even after my return to Germany.

Professor Tawara made a pioneering scientific contribution to the elucidation of the conduction system of the heart. His journey to this point was long and painful: even before entering First Higher School (Japan), he began to study German. He continued these language studies in high school and Tokyo University.

In 1903, Tawara traveled to Germany and decided to work at the University of Marburg with Professor Ludwig Aschoff, who received a call there in the same year. After initial difficulties of collaboration, the cooperation developed in a good direction. This was also because of the fact that Aschoff was a gifted scientist and university teacher: more than 400 of Aschoff's own papers were published, and more than 1000 publications by his students can be attributed to his work and initiative. Aschoff had many students from Japan: In the early 20th century, 23 of 26 chairs at Japanese pathological institutes were occupied by students of Aschoff.

Aschoff's early student Sunao Tawara was one of the particularly outstanding scientists at his chair: Tawara discovered the node named after him and Aschoff, which he called the cardiomotor center of the heart in his “Preliminary Communication” on the “Topography and Histology of Bridging Fibers” in 1905. In 1906, he published a detailed description of his findings in the book “Das Reizleitungssystem des Saugetierherzens.” In the same year, the book “Die heutige Lehre von den pathologisch‐anatomischen Grundlagen der Herzschwache,” published jointly by Aschoff and Tawara, appeared.

Tawara has to share the internationally valid name designation of the AV node with his academic teacher Aschoff (Aschoff‐Tawara node). The research was continued and secured after Tawara's return by follow‐up work of Aschoff and his German student Wilhelm His (Junior) until 1908. The two described the AV node (Aschoff‐Tawara node) located in the myocardial bundle and had thus proved the atrioventricular node to be a secondary pacemaker of the heart. Only then was the significance of Tawara's scientific work finally secured.

Tawara's discovery of the divisions of the atrioventricular node into two main bundles is quite different. Tawara described the histology and the macroscopic and microscopic anatomy of the atrioventricular muscle connections and their division into the two main bundles in the animal heart as well as in the human heart. He presented with extraordinary precision the anatomy of the excitation conduction system starting from the His bundle to the Purkinje fibers. These scientific works bear Tawara's name alone (Tawara's thigh).

When Tawara returned to Japan in 1906, the joint book by him and his academic supervisor quickly attracted wide attention. He went on to a rapid academic career and was a professor at Kyushu University for many years. Aschoff remained loyal to Japan and visited the country and its many students in 1926.

The close collaboration between Aschoff and Tawara now goes back some 120 years. It is an expression of a fruitful synthesis between a German and a Japanese, which could also take place in this or a similar way in modern times. Curiosity for new things, but also overcoming cultural and linguistic barriers, patience and an unconditional will to push things forward against resistance of all kinds‑all this has not changed in these 120 years‑despite the Internet and translation software. In this sense, the work of Tawara and Aschoff is an incentive for the younger generation to follow the two scientists on their strenuous journey to better understand the world. This applies to the bilateral and multilateral scientific enterprise of all disciplines in a global context, but especially to German‐Japanese scientific cooperation. Medical challenges abound‑medical progress not only leads to better treatment options, but often uncovers more questions than were previously answered. So there is a lot to be done. Of course, this also applies to cardiology.

Posthumously considered, both scientists were early ambassadors for a global world that, in addition to scientific curiosity, also had the goal of improving the quality of life of people. May as many as possible follow this primordial universal goal of the two!

I wish the Japanese Heart Rhythm Society much success for your event.

## GREETING ADDRESS FROM THE MEDICAL DEPARTMENT OF MARBURG UNIVERSITY

Prof. Dr. Denise Hilfiker‐Kleiner, Secretary of Dr. Hilfiker Kleiner (Dean), Dekanin dekanin.fb20@uni-marburg.de

Dean of the Faculty of Medicine at Philipps‐Universität Marburg, Professor of Cardiovascular Complications in Oncological Therapies, Philipps‐Universität Marburg

Members of the Japanese Heart Rhythm Society

As the Dean of and on behalf of the Medical Faculty at the Philipps University of Marburg, I would like to extend my special congratulations on the 150th birthday anniversary of Professor Dr. Sunao Tawara.

As a molecular cardiologist, I am aware of the great importance that the discovery of the atrioventricular node and its connections in the excitation conduction system has substantially contributed to understand the physiology of the heart and pathophysiological alterations in heart disease. For this reason, the President of the University, the Lord Mayor of the City of Marburg, the CEO of our University Hospital, Dr. Weiß and myself named two streets or squares last December after Tada Urata, the first Japanese physician to receive a doctorate in Marburg, and after Sunao Tawara and his teacher Ludwig Aschoff, the discoverers of the cardiac conduction system. In the future, Tada Urata Square near the Central Medical Library and the Tawara Aschoff Node between the Clinical Center and the Natural Sciences Preclinical Campus will bring their names to the attention of all students, patients and visitors. In 2027 the Philipps University in Marburg will celebrate its 500th anniversary, the names of Tada Urata and Sunao Tawara will also be duly mentioned as witnesses to the long‐standing relations between Japanese and German scientists. I would be pleased if these fruitful scientific relations would continue in the future.

## GREETING ADDRESS TO THE JAPANESE HEART RHYTHM SOCIETY

Dr. Gerhard Aumüller, Prof. em. of Anatomy and Medical History, Faculty of Medicine at Philipps‐Universität Marburg, aumuelle@staff.uni-marburg.de

Dr. Harumi Murata, Psychiatrist, Marburg, harumi59@yahoo.de

Ladies and Gentlemen,

Dear Colleagues,

It is our great honor to send a greeting address to the Japanese Heart Rhythm Society on the occasion of the 150th birthday of Professor Dr. Tawara Sunao on July 5, 2023. With the discovery of the atrioventricular node of the cardiac conduction system, Tawara not only founded a new era in cardiology and the physiology of the heart, but at the same time established close ties with the Philipps University of Marburg through contact with his “doctoral supervisor” and teacher, Professor Dr. Ludwig Aschoff.

It is an interesting historical coincidence that a few years after the transfer of the Electorate of Hesse with its State University of Marburg to the Kingdom of Prussia in 1866, in Japan during the Meiji era from 1868 to 1912, the Imperial University of Tokyo was founded in 1886. Already from 1880 to 1888, the later Marburg anatomy professor Josef Disse had written the first Japanese textbook of anatomy in Tokyo. This laid the foundation for close cooperation between Japanese scientists and their German colleagues. Examples of the fruitful relations also later are the collaboration of the famous professors Kitasato Shibasaburo (1852–1931) and his student Kitashima Taichi (1870–1957) with Emil von Behring (1854–1917) in Marburg. It was also Behring who brought the young pathology professor Ludwig Aschoff (1866–1942) to Marburg.

From Easter 1903 to mid‐July 1906, the young physician Tawara Sunao worked in his Marburg Pathological Institute. After studying medicine at the University of Tokyo, he first continued his training in Japan and was then to begin his dissertation in Germany with a scholarship from his adoptive father. With tremendous diligence, great technical skill and accurate powers of observation, he succeeded, after many setbacks, in fundamentally clarifying the structure of the cardiac conduction system, which had previously been inadequately studied. The discovery of the sinus node by Arthur Keith and Martin Flack (1907) then provided the basis for the interpretation of the electrophysiology of the heart by Willem Einthoven (1903) and Thomas Lewis (1915).

At about the same time, from 1903 to 1905, the Japanese physician Urata Tada (1873–1936) was working on her doctoral dissertation in the eye clinic located directly next to the Pathological Institute. She had been a student of Prof. Kitasato Shibasaburo in Tokyo and was the first Japanese woman in Germany to earn her doctorate in Marburg. Later she established herself as an ophthalmologist in Japan and founded and managed a clinic there.

Last year, the city of Marburg paid special tribute to the memory of these two eminent Japanese physicians. In a ceremony on December 6, 2022, the Lord Mayor of the City of Marburg, Dr. Thomas Spies, the President of Philipps University, Prof. Dr. Thomas Nauss, the Dean of the Department of Medicine, Prof. Dr. Denise Hilfiker‐Kleiner, the City Council President, Dr. Elke Neuwohner, together with other speakers and the two signatories, named two streets in the area of the University Hospital after the two Japanese scientists: The square in front of the Central Medical Library now bears the name “Tada Urata Square,” and the connecting street between the hospital and the natural science and medicine departments was named “Tawara Aschoff Node.”

In this context, we would also like to remember Professor Dr. Suma Kozo (1932–2020), who pointed out the scientific importance of Professor Tawara in several publications and did much to cultivate his memory. To commemorate Tawara's work, Prof. Suma presented a large painting by the Japanese artist Yoshino Toshio as a gift to Prof. Dr. Horst F. Kern, then Dean of the Department of Medicine, Director of the Institute of Clinical Cell Biology, and later President of Philipps University, at the 1st International Aschoff‐Tawara Symposium in early July 1999. It still resides in the former Institute of Pathology, the place where Tawara and Aschoff worked, the site of their most significant discovery. At that time, Prof. Suma, in an exceedingly generous manner, provided an original drawing by Tawara depicting muscle fibers of the AV node for a commemorative vitirine that the undersigned (GA) established in Marburg to commemorate the collaboration of Japanese and German investigators (see Figure).

We hope that the connection between Japanese and German scientists will continue to be maintained and developed in the spirit of international understanding and medical research for the benefit of patients. Your anniversary event is a hopeful sign for this.

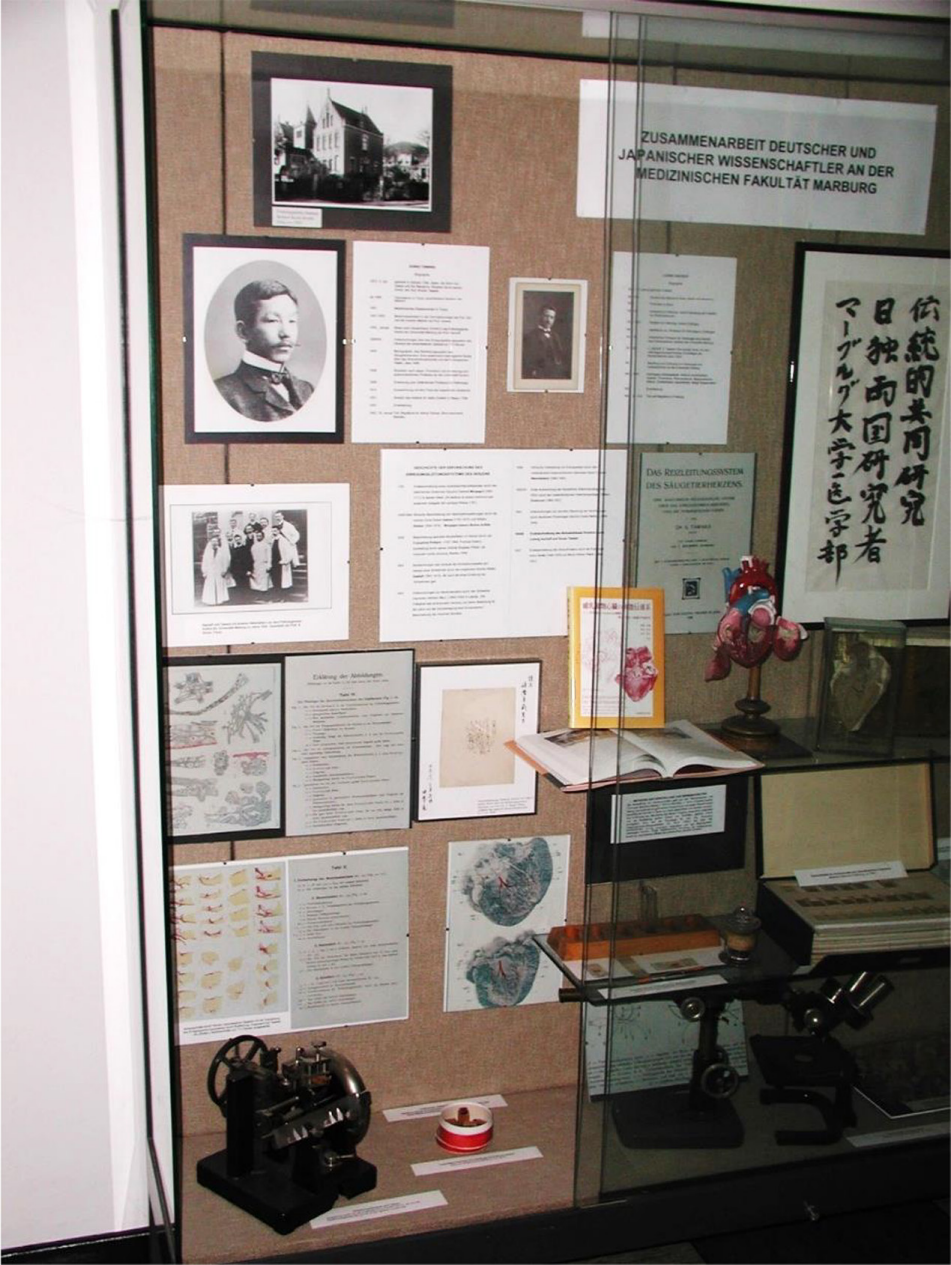

## SUNAO TAWARA AND KOZO SUMA

MUNEHIRO SHIMADA, Appointed Prof. Dokkyo Medical University. frx9a-m@east.cts.ne.jp

This year (2023) marks the 150th anniversary of TAWARA's Birth (1873) and a commemorative collection in English is planned to be published. It is my great honor and pleasure to be allowed to write a short story about SUNAO TAWARA and KOZO SUMA.

One Saturday morning about 40 years ago, Prof. KOZO SUMA has called me and said.“Why not work with me?!”He showed extraordinal Intention to translate the original work of SUNAO TAWARA into English: “Das Reizleitungssystem des Säugetierherzens” (1906). At that time, only one original copy remained in Japan. TAWARA's world‐famous achievement was on the verge of being buried in the darkness of the medical history. There was even an anecdote that TAWARA was a Spanish in a lecture at an american university. Prof. SUMA was passionate enough to translate the German original monograph, first into Japanese and then into English.

TAWARA worked very hard day and night at the Department of Pathology, led by Prof. Ludwig Aschoff, Prof. of Pathology at the Philipps‐ University of Marburg. TAWARA studied using just a single optical microscope with his surprisingly great insight, for example, the sketch of minute analysis of the fiber arrangements of a dog A‐V Node, that of the continuation from A‐V Node to His Bundle, the mode of the conduction system into the right and the left bundle branches, spreading into Purkinje Fibers. Consequently, he found all of the conduction system of the mammalian hearts except for S‐A Node (Keith and Flack, 1907) and wrote a monograph of approximately 200 pages in German. In 1990, Prof. SUMA completed the Japanese translation of the monograph and published by Maruzen Corporation under the names of Kozo Suma, Munehiro Shimada, and Tatsuo Shimada.

Prof. SUMA and I continued together to translate the work into English, 2–3 h a day, 2–3 times a week, altogether 500 times . In 2000, the work was published by Imperial College Press, London, with a preface by Prof.R.H. Anderson, and further donated to major libraries around the world. Now, It is printed in the United States with a white hard cover. In this way, TAWARA ‘s breakthrough achievement was finally preserved in English in the history of medicine.

Six letters written by TAWARA to his mentor Aschoff were incidentally found by Prof. Jürgen Aschoff, eldest son of L. Aschoff, in the attic of his house in Freiburg. I remember still very vividly, when those letters were sent to me. It was clear, that Prof. L. Aschoff had the intension to preserve TAWARA's great achievement in the medical history. On the contrary, Aschoff's letters replying TAWARA could not be found. The Soviet soldiers might have burned them to keep their warm. At the farewell party for TAWARA, Aschoff said that he was so taciturn that he could not know what TAWARA was really thinking. However, the teacher‐student relationship after that was so marvelous that Aschoff visited TAWARA's house in Fukuoka Japan in 1924.

Although there is a document stating that TAWARA graduated from the same German language school as Tatsukichi Irisawa, first professor of internal medicine at the University of Tokyo School of Medicine (graduated in 1889). the school was closed a short time later and the list of graduates has been lost.

Prof. SUMA, after graduation of the university of Tokyo, joined the Second Department of Surgery (Prof. Seiji Kimoto) and was assigned to ME group of artificial internal organs, and in 1963, with cooperation of Prof. Tatsuo Togawa, Prof.of engineering, he made the first Japan‐made implantable pacemaker and applied it in a patient with complete AV‐block who had fainting spells repeatedly. This was two years before the German‐made pacemaker used clinically in Germany. They say, that TAWARA is the father of pacemaker.

Mr. Naoki Suma, eldest son of Prof. SUMA, looking back on his father and wrote: “My father was the type of person who tells what should be done as a human being with his back. Prof. SUMA took good care of those in needs and hardships, stood quietly by their sides, with a balance of moderate strictness and a good sense of discretion with a little bit of mischievousness. My father had a strong presence even without speaking in each space.” It is quite an apt description also to me. While we were doing the work, I met a crisis in my life. At that time, Prof. SUMA, supported and guided me surprisingly well. Prof. SUMA was really a benefactor for me too. Also, there is an anecdote, that a nurse who was taking care of Prof. SUMA during his dying moments said to his son, Mr. Naoki: “When I am taking care of Prof. SUMA, I felt as if I was healed too.” Needless to say, his outstanding personality earned him the respect of many people. Prof. SUMA was a man of warmth and wisdom who has informed TAWARA's great achievement to the general public. Prof. SUMA's last word to me was: “It was really a big project, is not it!!”

Prof. SUMA passed away on November 9, 2020 (resulting in pancreatic cancer). It seems to me that Prof. SUMA's character was somewhat similar to that of TAWARA. I would like to express my sincerest condolences.

Thank you very much.

Photos 1 and 2

**PHOTO 1:**
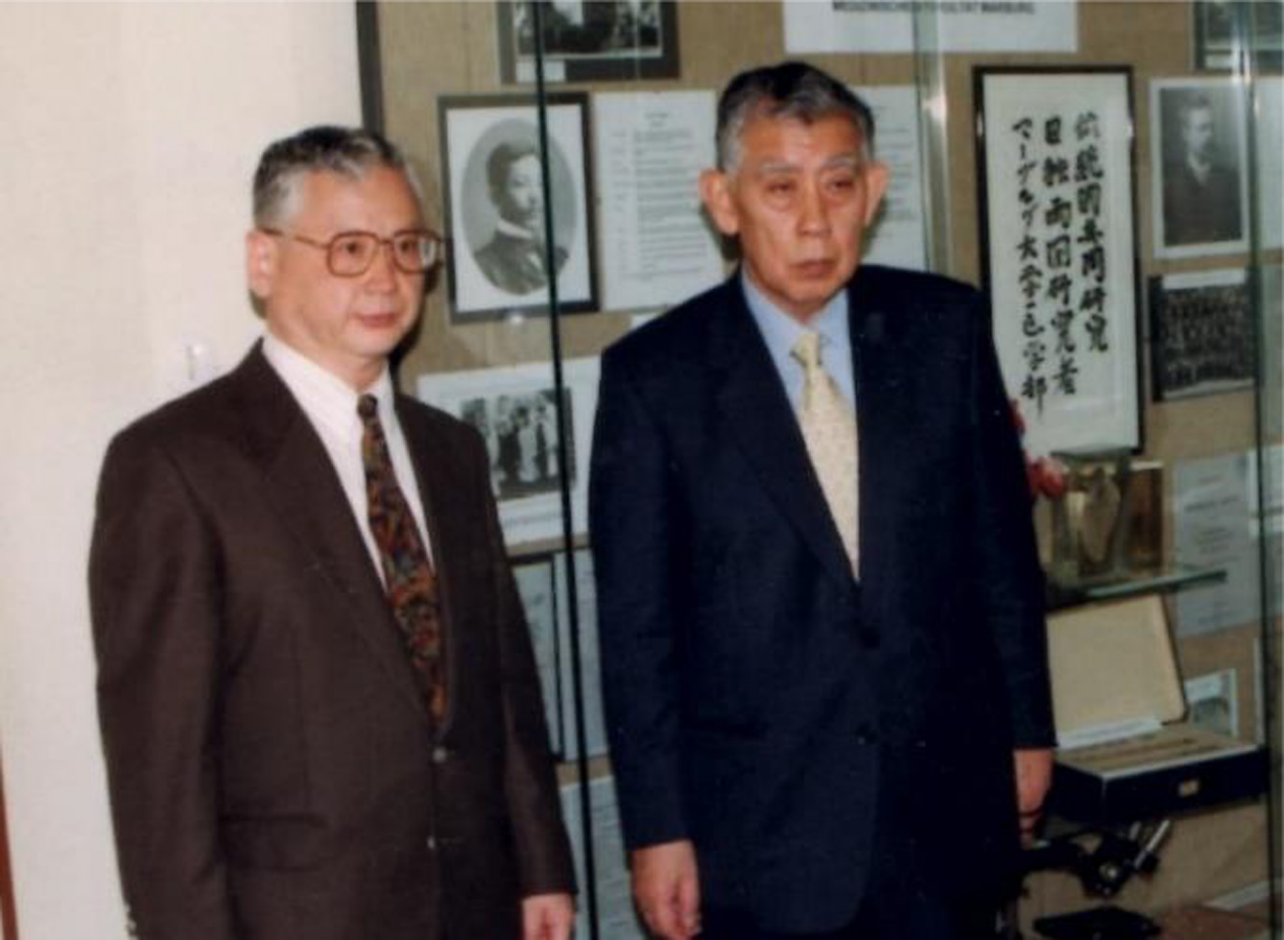
In front of the SUNAO TAWARA Memorial Showcase installed at the University of Marburg. Right: Prof. Kozo Suma, Left: Munehiro Shimada.

**PHOTO 2:**
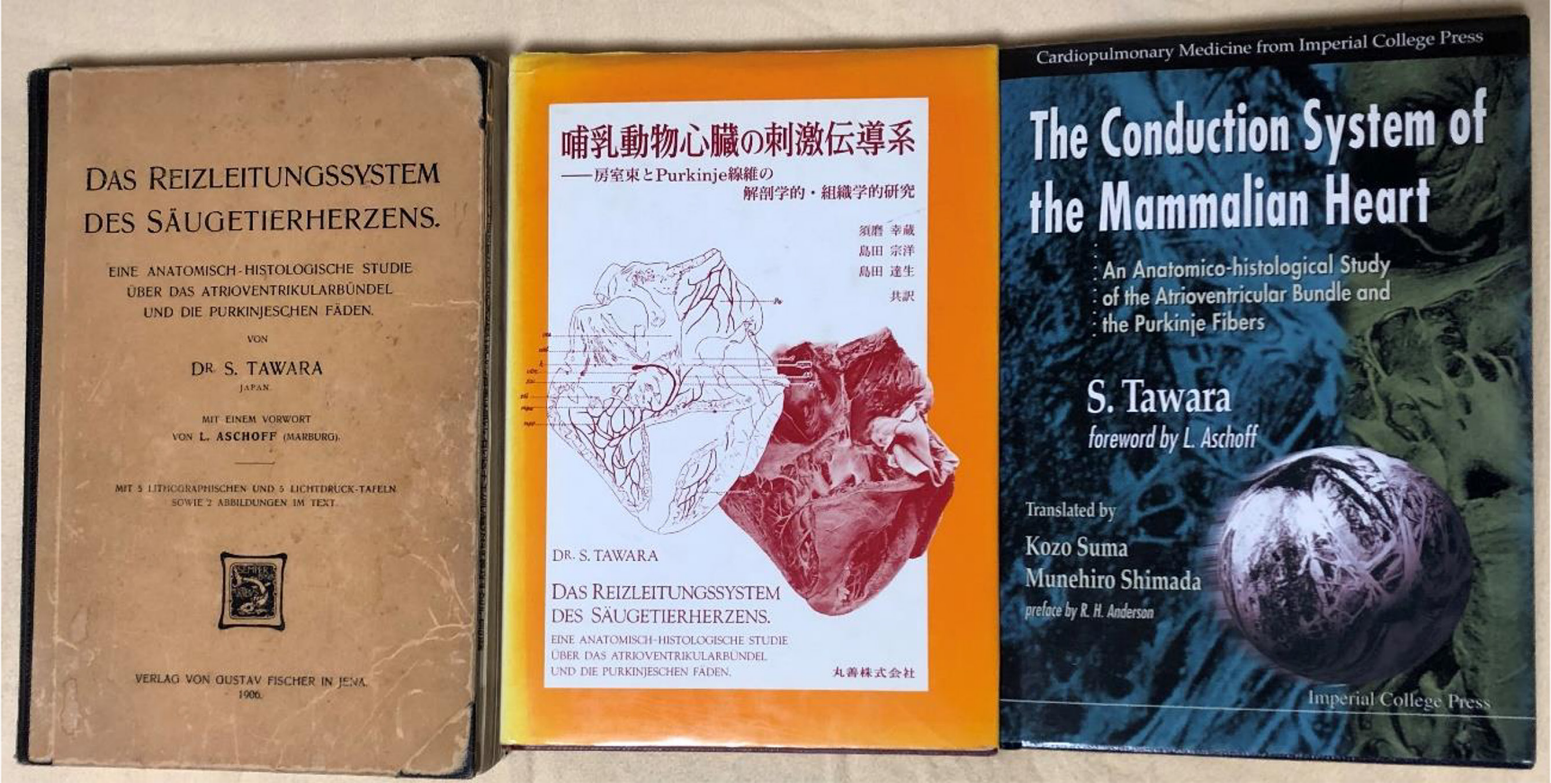
From left to right, covers of the reprint of TAWARA's original work, the Japanese translation and the English translation.

## CELEBRATING SESQUICENTENNIAL ANNIVERSARY OF PROFESSOR SUNAO TAWARA

Shumpei Mori, MD, PhD, Kalyanam Shivkumar, MD, PhD

UCLA Cardiac Arrhythmia Center, UCLA Health System, David Geffen School of Medicine at UCLA, Los Angeles, CA, USA

**Correspondence**

Kalyanam Shivkumar, MD, PhD, UCLA Cardiac Arrhythmia Center, UCLA Health System, David Geffen School of Medicine at UCLA, 100 UCLA Medical Plaza, Suite #660, Los Angeles, CA 90095, USA.

Email: kshivkumar@mednet.ucla.edu, smori@mednet.ucla.edu

It is our great privilege to have an opportunity to send this special message to Japanese Heart Rhythm Society, for its celebration of the sesquicentennial anniversary of the birth of Professor Sunao Tawara (1873–1952). Professor Tawara is credited with the discovery of the atrioventricular node, eponymously referred to as Tawara's node. Moreover, the modern concept of the entire atrioventricular conduction system was comprehensively established by Tawara with surprisingly precise physiological assumptions. His enormous and meticulous effort on both micro‐ and macroscopic observations eventually enabled him to “draw” one of his classic illustrations of the atrioventricular conduction system in his monograph.^1^ Tawara's anatomical work has provided the foundation for current clinical practice of cardiology. In 2023, we prepared a special panel (Figure) to celebrate 150th anniversary of his birth as a part of our UCLA Tawara‐McAlpine Cardiac Anatomy Project. This panel was created with images provided by Dr. Michiaki Murayama (Murayama Clinic), Dr. Kazunori Nakagawa (Kyushu University), Dr. Yoshinao Oda (Kyushu University), and Dr. Tatsuo Shimada (Oita University).

Wallace Arnold McAlpine (1920–2005) was a cardiac surgeon in Toledo, Ohio, United States. His classic Atlas with 1098 exceptional anatomical photographs and illustrations of the hearts was published in 1975.^2^ This beautiful Atlas received recognition throughout the world, including a Gold Medal at the International Book Festival in Leipzig, Germany. Uniquely, all the hearts were pressure‐perfused and fixed to prevent distortion. The hearts were fixed on the special rotational mount tripod and pictured at dedicated photo‐studio set at the basement of his house. This allowed him to record photographs of non‐distorted hearts with an attitudinal orientation. One of the important messages of this Atlas was that three‐dimensional cardiac anatomy should be shared and discussed in the setting of non‐distorted heart and viewed from clinically relevant directions. His entire collection counts more than 2500 panels of remarkable images of the heart, including unpublished ones. It is currently preserved in UCLA Cardiac Arrhythmia Center.

Inspired by Tawara and McAlpine, we have launched UCLA Tawara‐McAlpine Cardiac Anatomy Project in 2019 under the auspices of the larger Human Anatomy effort‐ The UCLA Amara Yad Project (https://www.uclahealth.org/sites/default/files/documents/Amara‐Yad‐Project‐UCLA.pdf). We believe that revisiting the anatomy of the conduction system using the approach established by McAlpine is a necessary step for clinicians to appreciate the crucial and elegant discovery made by Tawara.^3^, ^4^ We believe these approaches will provide novel insights into the clinical cardiac anatomy of the conduction system. Through this project, we are trying to establish comprehensive digital educational archive of three/four‐dimensional cardiac anatomy that can be shared everywhere in the world.^5^ Furthermore, based on the three/four‐dimensional anatomical knowledge, we aim to clarify physiological and pathological excitation of the human heart and develop sophisticated educational/diagnostic/therapeutic tools to improve clinical practice.

Our profound admiration and respect for Tawara's discovery has helped us to foster a tight connection between UCLA Cardiac Arrhythmia Center and Japan! Since 2002 when UCLA Cardiac Arrhythmia Center was founded, we have welcomed 15 visiting scholars from all around Japan through our UCLA‐Japan Electrophysiology Program. We hope our connection with Japan thrives further, and outcomes from UCLA Tawara‐McAlpine Cardiac Anatomy Project in the coming years helps improve our clinical practice to help patients all over the world.

REFERENCES1

Tawara
S
. The Conduction System of the Mammalian Heart. An Anatomico‐histological Study of the Atrioventricular Bundle and the Purkinje Fibers. London: Imperial College Press; 1906.2

McAlpine
WA
. Heart and coronary arteries. New York: Springer‐Verlag; 1975.3

Mori
S
, 
Hanna
P
, 
Bhatt
RV
, 
Shivkumar
K
. The atrioventricular bundle: a sesquicentennial tribute to professor Sunao Tawara. J Am Coll Cardiol EP. 2023;9:444–447.10.1016/j.jacep.2022.11.011367524814

Mori
S
, 
Moussa
ID
, 
Hanna
P
, 
Shivkumar
K
. Veiled anatomy of the tricuspid valve perimeter: what the Interventionalist must know…but cannot see!
J Am Coll Cardiol Intv. 2023;16:614–616.10.1016/j.jcin.2022.11.009367649165

Mori
S
, 
Shivkumar
K
. In: 

Shivkumar
K

, editor. Atlas of cardiac anatomy. Cardiotext Publishing. Volume 1. Hopkins, Minnesota: Anatomical Basis of Cardiac Interventions; 2022.

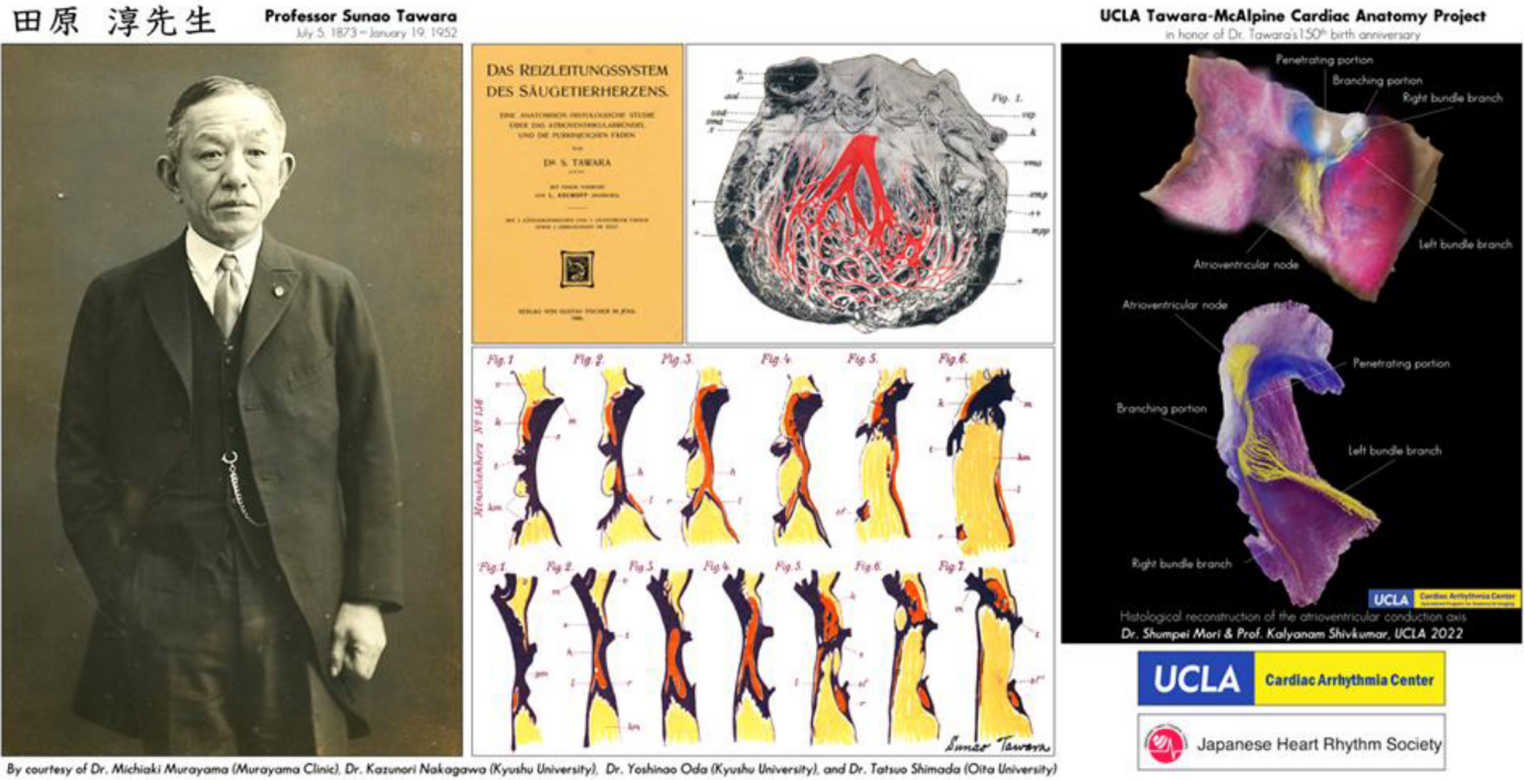

## TAWARA'S FOOTMARK LEADING THE MODERN CONCEPT OF ATRIOVENTRICULAR NODAL REENTRANT TACHYCARDIA

Yoshiaki Kaneko^1,2^, MD, PhD

From

^1^Department of Medicine, Tokorozawa‐daiichi Hospital, Saitama

^2^Department of Cardiovascular Medicine, Gunma University Graduate School of Medicine, Maebashi

**Correspondence**

Yoshiaki Kaneko, MD, PhD, Department of Medicine, Tokorozawa‐daiichi Hospital, 1559–1 Shimo‐yasumatsu, Tokorozawa, Saitama 359–0024, Japan.

E‐mail: kanekoy@gunma-u.ac.jp, yoshiakik33922@gmail.com

From the historical view, initial descriptions regarding today's paroxysmal supraventricular tachycardia can be found in the early 19th century, but the genesis of the arrhythmia has remained unclear a while. Actually, the great discoveries by Dr Sunao Tawara^1^ not only contributed an establishment of cardiac specialized conduction system including atrioventricular (AV) node, but also provided the anatomical basis leading to understandings of the current concept of AV nodal reentrant tachycardia.

Soon after his discovery, a reentrant phenomenon in the AV node was experimentally reproduced by Miles.^2^ Then, as can been seen from naming of tachycardia called paroxysmal *nodal* tachycardia described in the first half of the twentieth century,^3^ AV node seems to be already considered as the origin of the tachycardia before the first conceptualization of dual AV nodal physiology on the basis of experimental study performed by Gordon Moe and his collaborators.^4^ Shortly thereafter, based on the observation of discontinuous AV conduction during clinical eletrophysiologic studies, the existence of dual AV nodal conduction was also confirmed in humans and was proposed as the electrophysiological substrate of slow‐fast AV nodal reentry.^5^ However, at that time, the precise location of the slow pathway (SP) was uncertain. The first sign that the atrial end of the SP was away from the fast pathway (FP) was recognized during a clinical electrophysiologic study, when the earliest site of atrial activation during retrograde conduction over the SP was recorded at the proximal coronary sinus (CS).^6^ This simple, but innovative observation opened the way to the cure of AV nodal reentrant tachycardia by the surgical dissection of the SP,^7^ followed by its selective catheter ablation,^8^ keeping antegrade conduction intact over the FP. Following these clinical evidences suggesting the location of the SP at the posteroseptum, Inoue and Becker anatomically confirmed the presence of right posterior extension extending from the compact node into the right‐sided posteroseptum as a substrate responsible for the SP.^9^ They also pointed out the presence of left posterior extension extending into left‐sided posterosetpum in some cases that was thereafter confirmed to function as a variant of the SP, in clinical investiations.^10^ (As a side note, Japanese physicians often use terms of right [or rightward] or left [or leftward] *inferior* extension according to the descriptions by Jackman.^11^)

However, as described by Inoue and Becker, in their report,^9^ both of right and left posterior extension was not firstly discovered by them, but was already described in 1906 by Tawara.^1^ These posterior extensions have not received much attention since the exact location of the SP came into the spotlight, and it seems almost as if the descriptions regarding those by Tawara have been forgotten completely. This appears to result from the diversities of the medical sciences. According to the descriptions in Inoue's paper, “*Tawara's 1906 epic work “Das Reitzleitungssystem des Säugetierherzens” clearly states that a small, parallel‐oriented bundle of fibers originates from the node to run posteriorly approximately to the anterior region of the CS, where it connects with the usual atrial fibers*.^*1*^ *Tawara's famous plates, composed of meticulously reconstructed drawings of microscopic sections, beautifully illustrate these extensions and actually show that Tawara himself had already noticed that these bundles of fibers diverted rightward and leftward*.” Tawara's works also seem to teach clinical researcher an importance of the meticulous observations that might be apparently innocent at that moment, and descriptions of those to posterity.

REFERENCES1

Tawara
S
. Das Reitzleitungssystem des Säugetierherzens: Eine anatomisch‐histologische Studie über das Atrioventrikularbündel und die Purkinjeschen Fäden. Jena, Germany: Gustav Fischer; 1906. p. 135–136.2

Mines
GR
. On dynamic equilibrium in the heart. J Physiol (London). 1913;46:349–383.16993210
10.1113/jphysiol.1913.sp001596PMC14204303

Cowan
J
. Some disturbances of the rhythm of the heart. Br Heart J. 1939;1:3–26.18609805
10.1136/hrt.1.1.3PMC5034034

Moe
GK
, 
Preston
JB
, 
Burlington
H
. Physiologic evidence for a dual A‐V transmission system. Circ Res. 1956;4:357–375.13330177
10.1161/01.res.4.4.3575

Denes
P
, 
Wu
D
, 
Dhingra
RC
, et al. Demonstration of dual A‐V nodal pathways in patients with paroxysmal supraventricular tachycardia. Circulation. 1973;48:549–555.4726237
10.1161/01.cir.48.3.5496

Sung
RJ
, 
Waxman
HL
, 
Saksena
S
, 
Juma
Z
. Sequence of retrograde atrial activation in patients with dual atrioventricular nodal pathways. Circulation. 1981;64:1059–1067.7285296
10.1161/01.cir.64.5.10597

Ross
DL
, 
Johnson
DC
, 
Denniss
AR
, 
Cooper
MJ
, 
Richards
DA
, 
Uther
JB
. Curative surgery for atrioventricular junctional ("AV nodal") reentrant tachycardia. J Am Coll Cardiol. 1985;6:1383–1392.4067119
10.1016/s0735-1097(85)80229-18

Jackman
WM
, 
Beckman
KJ
, 
McClelland
JH
, 
Wang
X
, 
Friday
KJ
, 
Roman
CA
, et al. Treatment of supraventricular tachycardia due to atrioventricular nodal reentry by radiofrequency catheter ablation of slow‐pathway conduction. N Engl J Med. 1992;327:313–318.1620170
10.1056/NEJM1992073032705049

Inoue
S
, 
Becker
AE
. Posterior extensions of the human compact atrioventricular node: a neglected anatomic feature of potential clinical significance. Circulation. 1998;97:188–193.9445172
10.1161/01.cir.97.2.18810

Otomo
K
, 
Okamura
H
, 
Noda
T
, 
Satomi
K
, 
Shimizu
W
, 
Suyama
K
, et al. "left‐variant" atypical atrioventricular nodal reentrant tachycardia: electrophysiological characteristics and effect of slow pathway ablation within coronary sinus. J Cardiovasc Electrophysiol. 2006;17:1177–1183.16978247
10.1111/j.1540-8167.2006.00598.x11

Lockwood
DJ
, 
Nakagawa
H
, 
Dyer
JW
, 
Jackman
WM
. Electrophysiological characteristics of atrioventricular nodal reentrant tachycardia: implications for the reentrant circuits. In: 

Zipes
DP

, 

Jalife
J

, editors. Cardiac electrophysiology: from cell to bedside. Philadelphia: Elsevier; 2014. p. 767–787.
